# Comparison of cortisol levels in patients with vasovagal syncope and postural tachycardia syndrome

**DOI:** 10.12669/pjms.38.1.4122

**Published:** 2022

**Authors:** Humaira Fayyaz Khan, Shazadi Ambreen, Hammad Raziq, Azmat Hayat

**Affiliations:** 1 Dr. Humaira Fayyaz Khan, MBBS, FCPS Physiology, MHPE, Department of Physiology, Riphah International University, Islamabad, Pakistan; 2 Dr. Shazadi Ambreen, MBBS, MPhil Physiology. Department of Physiology, Shifa Tameere-e-Millat University, Islamabad, Pakistan.; 3 Dr. Hammad Raziq, MBBS, MPhil Physiology. Department of Physiology, Riphah University, Islamabad, Pakistan; 4 Dr. Azmat Hayat, MRCP, FRCP. Fellow Electrophysiology, Armed Forces Institute of Cardiology, Rawalpindi, Pakistan

**Keywords:** Serum Cortisol, Head Up Tilt Test, Postural Tachycardia Syndrome, Syncope, Vasovagal Syncope

## Abstract

**Objectives:**

To compare the levels of cortisol in patients of vasovagal syncope (VVS) and postural tachycardia syndrome (POTS).

**Methods:**

A cross-sectional analytical study was conducted at Islamic International Medical College, Rawalpindi and Electrophysiology Department at (AFIC). This study included 80 subjects, comprising of 35 patients in each group of vasovagal syncope and postural tachycardia syndrome and 10 healthy subjects. Patients with complaint of syncope was evaluated for vasovagal syncope and postural tachycardia syndrome using Head Up Tilt Test (HUTT. Blood samples of all the participants were taken and serum cortisol was analyzed using ELISA method. Results were analyzed on SPSS Statistics 21 using ANOVA with a p value of ≤0.05 regarded as significant.

**Results:**

Hormonal analysis shows that cortisol levels in the vasovagal, postural tachycardia syndrome and in control group was 153±16.7pg/ml, 160.17±pg/ml, and 69.65± 5.8pg/ml respectively. Cortisol levels were significantly higher in both vasovagal and POTS groups as compared to controls with a p-value of 0.04 and 0.023 respectively. However, there was no significant difference between vasovagal and POTS patients with p value 0.570.

**Conclusion:**

It is concluded from the study that cortisol responses of VVS and POTS were positive.

## INTRODUCTION

Sudden loss of consciousness with inability to maintain postural tone is known as syncope. This is due to cerebral hypo-perfusion for short duration of time on the average 12 seconds.^1^ Prevalence of syncope is 42% with incidence of 6% per annum.^2^ There are many causes of syncope such as Reflex, Cardiac, Neurological, Endocrinological and Psychiatric illnesses. The vasovagal syncope is the most frequent type of syncope which is mediated by orthostatic and emotional stress.^2^-^4^ Another type mimicking vasovagal syncope is POTS. It is a heterogeneous disorder that disturbs normal autonomic control.^5^,^6^ Neurally mediated reflex syncope (vasovagal syncope) is characterized by a distinct prodrome (usually lightheadedness, nausea, diaphoresis or visual changes) followed by a sudden loss of consciousness and rapid recovery. The hallmark of Postural Tachycardia Syndrome is persistent tachycardia (160 bpm or higher) in upright position with complaints of severe fatigue, exercise intolerance, palpitations, near syncope, lightheadedness, and dizziness.^4^ Since syncope impairs the work performance, managing the orthostatic intolerance will improve the functional capability of an individual leading to improving the quality of life and professional outcome.^7^

Head Up Tilt Test is widely use to examine neuro-cardiovascular orthostatic responses in patients with unexplained symptoms of orthostatic intolerance. It has specificity of nearly 90%.^8^ HUTT stimulates an orthostatic stress where hemodynamic monitoring can be done simultaneously.^9^ Positive tilt test gives the diagnosis of neurogenic syncope. However, negative tilt test does not rule out the neurogenic syncope.^9^,^10^ Literature shows that there are neuroendocrine causes behind vasovagal syncope and postural tachycardia syndrome resulting in orthostatic intolerance. Catecholamine, Serotonin, Renin, Angiotensin (I, II) and cortisol have been shown to play critical roles in regulation of circulation i.e. blood pressure, heart rate and vascular tone.^7^,^11^ To ours knowledge there is limited data regarding the levels of cortisol in the patients of vasovagal syncope and postural tachycardia syndrome. The present study aims at determining the association of cortisol levels with vasovagal syncope and POTS. This may provide additional information regarding the diagnosis of syncope.

## METHODS

It was cross-sectional analytical study conducted at the Islamic International Medical College (IIMC) Rawalpindi in collaboration with Electrophysiology Department, Armed Forces Institute of Cardiology (AFIC) during the period from 25^th^ August 2020 to 28^th^ December 2020. The approval from ethical review committee (Ref: Ripah/IRC/20/216, Dated: August 21, 2020) was sought. The patients were selected by random sampling. The study comprised a total of 80 patients with 35 patients in each group of vasovagal syncope and postural tachycardia syndrome diagnosed on head up tilt test. While 10 healthy subjects were included in the study as a control group. All patients were included in this study after taking informed consent. Wherever necessary, they were diagnosed and treated according to the guidelines and hospital protocols. Patients with uncontrolled hypertension, recent myocardial infarction, angina patients or diabetes with autonomic dysfunction were excluded from the study.

### Data collection procedure:

The patients were provided with full background information and their informed consent was taken. After taking consent, the sample was taken of the patients coming from the outpatient department wards of AFIC & NIHD. Demographic data regarding age, episode of syncope, BMI was also collected.Following physical examination data was collected.Blood pressure was monitored with sphygmomanometer with patient lying supine for five minutes. Responses of Ps, Pd, heart rate, MAP and PP were recorded from lying to standing and then back to lying as well as using HUTT procedures as explained in the following paragraphs and compared.

### Electrocardiography (ECG):

12 lead ECG was done in all the patients. With consideration of heart rhythm, duration of the QT interval, bundle branch morphology before HUT test.

### Echocardiography:

Echocardiography was done to rule out the Valvular lesions and left ventricular dysfunction (low Ejection fraction).

### Head up tilt test protocol:

The patient was asked to fast after 12 o’clock midnight. The test was carried out in a quiet and dimly lit room. ECG electrodes were placed on patient’s chest, while a blood pressure cuff was placed on one arm and an IV cannula in the other arm of the patient. After recording the blood pressure, pulse and baseline ECG in the supine position, restraining straps was placed over the patient and the table was tilled to 70-degrees with head up.

ECG and heart rate was continuously monitored and blood pressure was recorded after every three to five minutes. The test was stopped if the patient complained of feeling dizzy and/or if the blood pressure or heart rate dropped below a certain level. Those who did not experience presyncope or syncope during that period, were given sublingual nitroglycerin (0.3mg) and then again monitored for presyncope or syncope like symptoms. Patients developing symptoms of syncope, presyncope were labelled as positive tilt test. The patients who did not develop these symptoms at all, were labelled as negative HUTT patients while the Positive Head Up Tilt Test makes the diagnosis of vasovagal syncope.

In the same setting if the patient’s heart rate increased by 30 beats a minute (bpm) or more (40bpm in those aged 12 to 19) usually within 10 minutes of standing. And also if that increase continued for more than 30 seconds accompanied by other symptoms were suggestive of POTS.

Blood sample were collected just after completion of tilt table test by venipuncture technique after taking all the aseptic measures. Samples were centrifuged and serum was stored at -80° C. Serum Cortisol levels in all blood samples was determined by ELISA method.

### Statistical Analysis:

Data collected was analyzed by using SPSS version 25. The Chi square was applied to analyze the categorical data and Pearson correlation was applied to find the correlation of quantitative data and ANOVA was applied to find the association of cortisol with VVS and POTS.

## RESULTS

Descriptive data showed that mean age of vasovagal syncope patients was more as compared to POTS patients with a p-value of 0.025. No significant difference was found in gender and episode of syncope in either group of VVS or POTS (Table-I).

**Table I:** Baseline characteristics of VVS and POTS patients.

Characteristics	Vasovagal syncope	POTs	p-value
***Age (years)***	39.34±11.8	33±11.3	0.025*
***Gender (%)***			
Male	30	25	0.145
Female	5	10	
***Episodes of syncope (n)***	3±2.1	2.7±1.4	0.478
***Blood Pressure (mm Hg)***			
Systolic	120±7	125±5	n.s
Diastolic	60±5	65±5	
***BMI (Kg/m^2^)***	21.95±6	23.95±8	n.s

n.s: denotes not significant.

Results of the study shows no correlation of cortisol with increasing number of episodes or age of the vasovagal syncope patients with r- value of 0.05 and 0.096 respectively. It also shows no correlation of cortisol with number of episodes or age in patients suffering from POTs with r- value of 0.244 and 0.11 respectively as shown in Table-II.

**Table II:** Correlation of Cortisol levels with number of syncopal attacks and age in patients of vasovagal syncope and POTS.

Characteristics of patients	Cortisol levels			

	Vasovagal syncope		POTS	

	r value	p-value	r value	p-value
Number of Episode	0.05	0.79	0.24	0.17
Age	0.1	0.58	0.11	0.47

**Fig.1:**
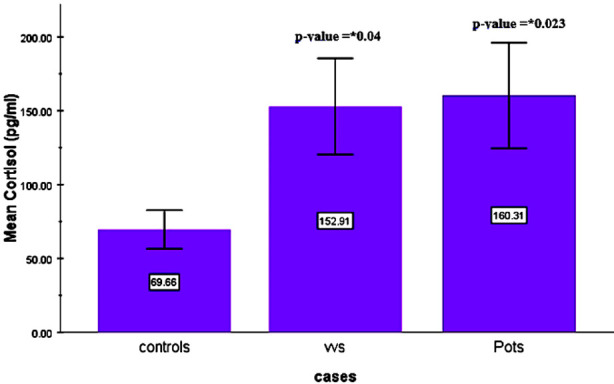
Comparison of mean serum cortisol levels of control group with VVS and POTS groups.

Significantly raised levels of cortisol in patients of VVS and POTS in comparison to healthy subjects with a p-value of 0.04 and 0.023 respectively is shown in Fig-1. But the difference between VVS and POTS was insignificant with a p-value of 0.94.

## DISCUSSION

The present study showed that serum cortisol responses of VVS and PTS are positive. This finding suggests its importance to rule out psychiatric, endocrinological or cardiac causes of orthostatic intolerance. Cortisol does have high levels in POTs as compared to VVS group. However, the difference is insignificant for the use in differential diagnosis. No correlation of age and syncopal episodes were found with serum cortisol in either groups of VVS and POTs.

Regulation of all systems such as cardiovascular, nervous, gastrointestinal or renal is dominated by hormones. Hormones such as Angiotensin, Renin, Aldosterone, Serotonin, Catecholamine and ANP are also involved in regulating the circulatory functions.^12^ This finding supports the present study. Another study while investigating the pathogenesis of neurogenic syncope demonstrated that perturbations in brain serotonin levels may result in pathophysiology of different disorders like neurocardiogenic syncope, carotid sinus hypersensitivity and orthostatic hypotension. Increased concentration of the serotonin results in the sympathetic withdrawal, causing hypotension and bradycardia leading toward syncope.^13^ Present study also suggests the role of cortisol to be associated with VVS and POTS. Increased serotonergic activity during head up tilt test was studied by Zarvalis et al., who evaluated the levels of cortisol and prolactin in the patients of syncope. He found that the level of prolactin and cortisol were raised significantly in positive head up tilt patients experiencing syncope which is in line with the results of present study.^14^ In POTS, most patients were found to harbor AT1R [angiotensin 1 receptor]. This might support the concept that antiadrenergic and AT1R autoantibodies may exert an impact on the POTS cardiovascular system.^11^ A study conducted by Lin et al., found raised levels of cortisol in children with POTs as compared to control group’s subjects. Raised cortisol levels were also found to be associated with severity of POT.^15^ Epinephrine and vasopressin were also found to be temporally associated with nausea related to tilt-induced syncope suggesting its possible association with syncope.^16^ This is in line with present findings which established that serum cortisol is associated with syncope. Association between c-AMP and VVS has been shown being driven by catecholamine changes.^17^ A study conducted to examine the confounding influences of sociodemographic factors on cortisol levels of stress patients. It documented a positive linear relationship between age and cortisol levels of the patients. Male cortisol levels were found to have higher than females in adult patients using hair samples.^18^ Another study showed the significant association of cortisol levels with increasing age.^19^ A study conducted to find cortisol differences in age and sex and its association with weight hip ratio in Swedish women showed significantly raised levels in older patients using salivary cortisol levels.^20^ While determining the impact of age on basal secretion of cortisol as well as on nutritional deprived state, they found significantly raised levels of cortisol in increasing age.^21^

## CONCLUSION

It is concluded from this cross sectional study that cortisol is raised in patients of vasovagal syncope and postural tachycardia syndrome.

### Authors’ Contribution:

**HFK:** Conceived, designed, interpretation of the data and is responsible for the integrity of the study..

**SA, HR:** Data collection and manuscript writing.

**AH:** Drafting the work and revising it critically for important intellectual content.
